# Levels of B-type natriuretic peptide in chronic heart failure patients with and without diabetes mellitus

**DOI:** 10.3892/etm.2012.760

**Published:** 2012-10-23

**Authors:** QIANG PENG, WEITONG HU, HAI SU, QING YANG, XIAOSHU CHENG

**Affiliations:** Department of Cardiology, Second Affiliated Hospital, Medical College of Nanchang University, Nanchang, Jiangxi 330006, P.R. China

**Keywords:** B-type natriuretic peptide, heart failure, diabetes mellitus, cardiac function

## Abstract

The current study aimed to observe the value of B-type natriuretic peptide (BNP) in chronic heart failure (CHF) patients with and without diabetes mellitus (DM). The study consisted of 559 CHF patients, including 276 patients with coronary heart disease, 234 with hypertensive heart disease and 49 with dilated cardiomyopathy. The subjects were divided into non-DM and DM groups which included 384 and 175 patients, respectively. Their New York Heart Association (NYHA) cardiac function degree and BNP levels were detected following admission. Other blood parameters, including fasting blood glucose and serum creatinine, were also collected. Left ventricular ejection fraction (LVEF) and the average thickness of the left ventricular wall (LVW) were detected by echocardiography. The total score of the heart failure (HF)-related parameters was evaluated for each patient according to age, hypertension, LVEF, LVW and NYHA degree. Additionally, BNP-score relation curves were constructed. The levels of BNP were significantly higher in the DM group compared with the non-DM group (1143.73±94.0 vs. 884.34±57.0 ng/l, P<0.05). Positive BNP-score relation curves were obtained for the DM and non-DM groups, but the curve of the DM group was notably steeper. As the patients with DM have significantly higher BNP levels at a similar HF score, DM history and fasting blood glucose should be taken into consideration when evaluating the value of BNP in HF.

## Introduction

Chronic heart failure (CHF) is an ongoing, progressive syndrome characterized by the impairment of cardiac function and an increase in neurohormonal activity (1). B-type natriuretic peptide (BNP) is a neurohormone primarily secreted by the cardiac ventricles which is now used as a diagnostic marker for CHF since it is easy to obtain and may be detected rapidly (2). BNP is a natural antagonist of the renin-angiotensin-aldosterone system (RAAS). It not only decreases systemic vascular resistance and central venous pressure, but also decreases blood volume and cardiac output (3). Since the levels of BNP are correlated with left ventricular dysfunction, measurement of BNP may help clinicians diagnose and evaluate heart failure (HF) rapidly. BNP measurement has become a routine part of CHF diagnosis (4).

It is well known that a high proportion of CHF patients have diabetes mellitus (DM), and the prognosis is worse for coronary heart disease patients who have DM than for those who do not (5,6). The Hoorn Study revealed that BNP levels are associated with markers of left ventricular diastolic function and changes in left ventricular mass. The association of BNP with left ventricular diastolic function appears to be particularly strong in individuals with DM (7). Other studies indicated that BNP was not only associated with left ventricular abnormalities but also with cardiovascular disease and mortality risk in DM patients (8,9). Therefore, when BNP is used to assess cardiovascular disease (CVD) risk in CHF patients, the presence or absence of DM should be taken into account (10,11). Meanwhile, certain studies demonstrated that the level of BNP in CHF patients with DM was higher than in CHF patients without DM, but provided no relevant evidence from strict control-based research (12,13).

The aim of the current study was to accurately investigate the influence of DM on the level of BNP in CHF patients, using a scaling method based on parameters including age, hypertension, LVEF, LVW and NYHA degree.

## Materials and methods

### Subjects

A total of 559 CHF inpatients from our hospital were enrolled in this study, including 276 patients with coronary heart disease, 234 with hypertensive heart disease and 49 with dilated cardiomyopathy diagnosed by coronary angiography. The study was conducted in accordance with the declaration of Helsinki and with approval from the Ethics Committee of Nanchang University. Written informed consent was obtained from all participants. The subjects were divided into a non-DM group and a DM group which included 384 and 175 patients, respectively. Blood samples for blood routine tests and biochemical tests of all subjects were drawn in the morning, following a 12 h fast. Plasma BNP was measured using a microplate luminometer reader (Centro LB 960; Berthold Technologies GmbH & Co., Bad Wildbad, Germany) and a Shionoria BNP kit (Shionogi Company, Osaka, Japan). As the minimum and maximum detectable concentrations were 0.1 and 4,000 ng/l respectively, BNP levels <0.1 ng/l were counted as 0.1 ng/l and BNP levels >4,000 ng/l were counted as 4,000 ng/l in this study. The above tests were performed in our clinical laboratory, and inter- and intra-batch coefficient of variations were controlled within 5.5 and 3.5%, respectively.

The left ventricular ejection fraction (LVEF) and the average thickness of the septal and posterior walls of the left ventricle (LVW) were detected by echocardiography. The cardiac function grade was assessed according to the NYHA criterion. Body weight, body height and seated blood pressure were measured and body mass index (BMI) was calculated. History of smoking and aspirin use were recorded.

Hypertension was divided into 3 grades based on blood pressure levels: grade 1 was systolic blood pressure (SBP) 140–159 mmHg and/or diastolic blood pressure (DBP) 99–90 mmHg; grade 2 was SBP 160–179 mmHg and/or DBP 100–109 mmHg; and grade 3 was SBP ≥180 mmHg and/or DBP ≥110 mmHg. Patients under DM treatment, or with fasting plasma glucose >7.0 mmol/l and/or 2 h postprandial plasma glucose >11.0 mol/l, were classified as having DM.

Based on the recorded data, parameters relevant to BNP were collected to calculate the total score for each patient (Table I). The highest score was 14 points and lowest was 4 points. The correlation curves of BNP with the score were constructed for the DM and non-DM groups.

### Statistical analysis

Statistical analysis was performed using SPSS 13.0 software package. All data were expressed as the mean ± standard deviation (SD). We used an independent sample t-test between the two groups and linear correlation analysis was performed. A p-value of <0.05 was considered to indicate a statistically significant difference.

## Results

Table II provides the general characteristics of the two groups. There were no differences in age, proportion of male subjects, number of smokers or blood pressure level between the DM and non-DM groups. However, white blood cell count, fasting blood glucose and the percentage of aspirin users in the DM group were higher than those in the non-DM group. No significant differences in the echocardiographic index, including LVEF and LVW, were identified between the two groups. However, the percentage of atrial fibrillation was lower in the DM than in the non-DM group.

**Figure 1:**
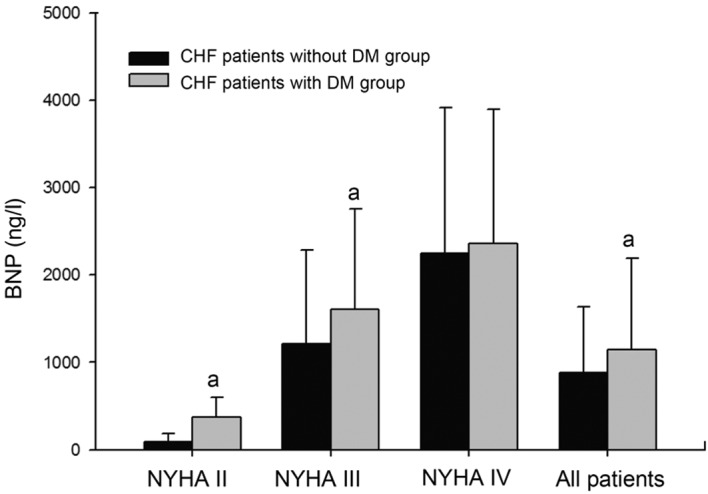
Plasma B-type natriuretic peptide (BNP) levels of all patients. ^a^P<0.05, compared with the non-DM group. CHF, chronic heart failure; DM, diabetes mellitus; NYHA, New York Heart Association.

Generally, the plasma BNP level was higher in the DM group than in the non-DM group (1143.7±94.0 vs. 884.3±57.0 ng/l, P<0.05). Based on the NYHA grade, the plasma BNP levels of the 3 DM subgroups were higher than those of the 3 non-DM subgroups. Statistical significance was observed with NYHA II (92.5±72.6 vs. 369.6±25.4 ng/l, P<0.05) and III (1603.8±152.6 vs. 1213.9±76.5 ng/l, P<0.05), but not with NYHA IV (2362.1±186.5 vs. 2249.9±157.0 ng/l, P>0.05; Fig. 1).

### Correlations between BNP and parameters

The linear regression analysis in 559 CHF patients revealed a negative correlation between plasma BNP level and LVEF (r=−0.511, P<0.05), but positive correlations between BNP level and NYHA (r=0.438, P<0.05), age (r=0.214, P<0.05) and LVW thickness (r=0.182, P<0.05).

**Figure 2:**
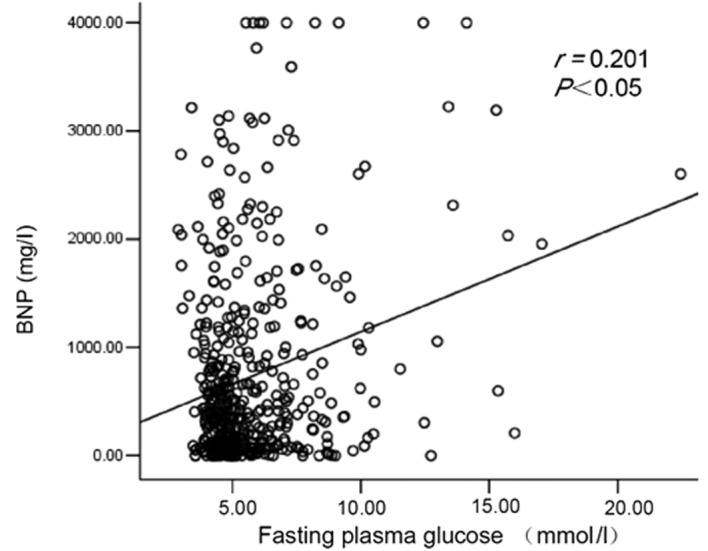
Correlation between plasma B-type natriuretic peptide (BNP) and fasting plasma glucose levels.

There was also a positive correlation between plasma BNP and fasting plasma glucose levels (r=0.201, P<0.05; Fig. 2).

### Scores of BNP-related parameters

**Figure 3:**
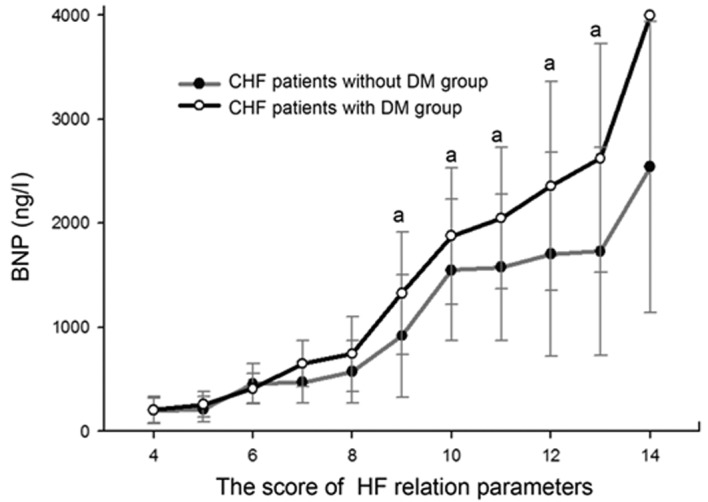
B-type natriuretic peptide (BNP)-score curves of the two groups. ^a^P<0.05, compared with the non-DM group. CHF, chronic heart failure; DM, diabetes mellitus; HF, heart failure.

The BNP-score curve clearly suggests that, for all patients, plasma BNP levels elevated as the score increased. The two curves separate at 7 points, after which the curve of the DM group is higher than the curve of the non-DM group. The plasma BNP levels of the subgroups with scores of between 9 and 13 points were significantly higher in the DM group than in the corresponding subgroups in the non-DM group. The positive correlation of plasma BNP level with the score was stronger in the DM group (r=0.523) compared with the non-DM group (r=0.436). The plasma BNP levels of the two patients with a total score of 14 points in the DM group were >4,000 ng/l (Fig. 3).

## Discussion

BNP is mainly secreted by ventricular muscle cells, and is directly related to ventricular volume change and ventricular wall tension. A number of studies have demonstrated that the level of plasma BNP may sensitively and specifically reflect the ventricular function and positively correlates with NYHA cardiac grades (14).

However, various factors, including obesity and renal insufficiency, may affect the BNP level in CHF patients. Recently, some studies showed that the level of BNP in CHF patients with DM was higher than in CHF patients without DM; however, most of those studies were simple intergroup comparisons rather than strict control-based research (12,13,15).

In the current study, a total score of the HF-associated parameters was evaluated for each patient according to age, hypertension, LVEF, LVW and NYHA degree. The purpose of this score was to accurately explore the influence of DM on the level of BNP in CHF patients at the same HF degree.

Our study not only demonstrated that the BNP level in CHF patients increased as the NYHA degree increased but also demonstrated that higher BNP levels were observed in the 3 NYHA subgroups with DM compared with the corresponding subgroups without DM. However, no significant difference was found in the NYHA IV subgroup. One reason for this may be the small number of subjects in that subgroup. Another possible reason is that the reported upper limit of BNP concentration was 4,000 ng/l, which underestimates the value of BNP in the DM subgroup, as there were more patients with BNP higher than 4,000 ng/l in the DM subgroup.

In this study, a BNP-score curve was introduced for quantitative evaluation of the extent of HF. Positive correlations between the score and BNP levels were observed in the DM group and in the non-DM group. The two curves began to separate at 7 points, after which the curve of the DM group was persistently above that of the non-DM group. Among the subgroups with scores of between 9 and 13 points, the plasma BNP levels were significantly higher in the DM group than in the corresponding subgroups in the non-DM group. These results further demonstrate that DM is a promoting factor for higher plasma BNP levels in CHF patients. Research has shown that BNP levels of coronary heart disease patients with DM are significantly high compared with those of coronary heart disease patients without DM.

The underlying mechanism for the higher BNP level in CHF patients with DM is not clear; however, it may involve an increase in BNP formation and a decrease in degradation (21).

A previous study has suggested that the kidneys may secrete natriuretic peptides that aid BNP degradation, such as neutral endopeptidase (NEP) (17). In patients with renal impairment, NEP secretion declined and BNP degradation subsequently decreased. Liu *et al* found that BNP levels in DM patients with renal failure were significantly higher than those in DM patients with normal renal function (18). Siebenhofer *et al* confirmed that plasma N-terminal pro-brain Natriuretic Peptide (NT-pro BNP) levels in DM patients with an abnormal urinary albumin excretion rate (UAER >20 μg/min) was higher than in DM patients with a normal UAER (19). Although UAER was not measured in the current study, as the parameters concerning renal function were similar between the DM group and the non-DM group, the elevated BNP may not be due to decreased excretion of BNP, and mostly due to increased formation.

Certain studies suggest that atrial fibrillation (AF) may be a cause of the increased BNP level (20). Since the DM group had a lower percentage of patients with AF than the non-DM group, AF is not a cause of the higher BNP level in the DM group. As the echocardiographic parameters such as LVEF and LVW were similar between the DM group and the non-DM group, the increased BNP formation unlikely to be caused by more severe cardiac remodeling.

Based on the positive correlation between the fasting glucose and BNP levels in this study, it is reasonable to speculate that higher plasma glucose levels may accelerate BNP secretion. Higher levels of plasma glucose may induce a hypertonic state, causing cell dehydration and an increase in blood volume. All these changes might increase the ventricular tension and therefore raise the BNP level. Moreover, in patients with DM, symptoms were often accompanied by vascular lesions more frequently found in coronary heart disease patients. Coronary heart disease may not only cause myocardial ischemia, hypoxia, necrosis and fibrosis, but also increase ventricular wall tensions and promote BNP secretion (14). Additionally, the DM patients often had cardiac autonomic dysfunction. A previous study has confirmed that an elevated BNP level is correlated with cardiac autonomic dysfunction (21).

Since the plasma BNP level was significantly higher in the DM group than in the non-DM group at the same HF score, when plasma BNP level is used to evaluate HF in a clinical environment, the factor of DM should be taken into consideration.

## Figures and Tables

**Table I:** Scoring of the HF-associated parameters.

	Score		
Related parameters	1	2	3
Age (y)	≤60	60–80	≥80
NYHA	II	III	IV
LVEF (%)	≥50	40–50	≤40
LVW (mm)	≤11	11–13	≥13
Hypertension	Grade 1	Grade 2	Grade 3

NYHA, New York Heart Association; LVEF, left ventricular ejection fraction; LVW, the average thickness of left ventricular wall.

**Table II:** Selected characteristics of the two groups.

	CHF with DM (n=175)	CHF without DM (n=384)
Age (years)	68.3±10.6	69.5±10.3
Male [N (%)]	102 (58.2)	233 (60.6)
BMI (kg/m^2)^	25.6±3.5^a^	23.3±3.4
Smoking history [N (%)]	62 (35.4)	125 (32.5)
Systolic blood pressure (mmHg)	142.3±28.6	139.2±24.7
Diastolic blood pressure (mmHg)	80.6±16.1	78.0±13.9
Aspirin use [N (%)]	50 (28.5)^a^	71 (18.4)
NYHA II:III:IV (N)	84:71:20	189:159:36
Atrial fibrillation [N (%)]	25 (14.2)^a^	90 (23.4)
Laboratory examinations		
WBC (10^9^/l)	8.4±4.8^a^	6.9±2.7
RBC (10^12^/l)	4.1±0.7	4.1±0.6
HB (g/l)	121.3±20.8	121.9±19.5
RDW (%)	14.0±1.3	13.9±1.3
PLT (10^9^/l)	189.8±68.1	184.5±65.1
CR (μmol/l)	92.8±48.7	89.3±41.2
FBG (mmol/l)	8.1±3.6^a^	4.87±0.8
Echocardiogram index		
LVEF (%)	55.6±12.9	57.2±13.2
LVW (mm)	10.1±1.3	10.2±1.4

CHF, chronic heart failure; DM, diabetes mellitus; BMI, body mass index; NYHA, New York Heart Association; WBC, white blood cell count; RBC, red blood cell count; HB, hemoglobin; RDW, red blood cell distribution width; PLT, platelet; CR, serum creatinine; FBG, fasting blood glucose; LVEF, left ventricular ejection fraction; LVW, the average thickness of left ventricular wall.

a P<0.05, compared with the non-DM group.
